# Cell-cycle machinery is critical in regulating uterine steroid hormone for embryo implantation and development

**DOI:** 10.1172/JCI186194

**Published:** 2024-11-15

**Authors:** Francesco J. DeMayo

**Affiliations:** Reproductive and Developmental Biology Laboratory, National Institute of Environmental Health Science, Research Triangle Park, North Carolina, USA.

## Abstract

Proper embryo implantation is necessary for a successful pregnancy. In this issue of the *JCI*, Aljubran et al. identified the cell cycle regulatory protein cyclin A2 (CCNA2) as a factor in supporting embryo implantation and embryo development. Endometrial stromal cells showed higher levels of CCNA2 in patients undergoing assisted reproductive technology who had successful pregnancies. CCNA2 expression correlated with stromal cell proliferation and the expression of steroid hormone receptors for estrogen (ESR1, also known as ERα) and progesterone (PGR). Notably, loss of *Ccna2* in mouse models resulted in infertility. The uteri of these mice were hypoplastic with reduced estrogen sensitivity, resulting in the disruption of stroma cell decidualization and loss of embryo viability after implantation. These findings demonstrate the importance of stroma cell proliferation in preparing the uterus for embryo implantation. They also identify CCNA2 as a coregulator of steroid hormone receptor signaling and suggest that impaired uterine stroma can underly early pregnancy loss.

## Endometrial stromal cells and embryo implantation

Successful embryo implantation is a result of the embryo attaching to the uterine epithelium during a defined window of receptivity. It also requires the endometrial stromal cells to undergo a differentiation process called decidualization (1). The decidualized stromal cells nurture and protect the implanting embryo and allow for appropriate placentation (2). It is hypothesized that the decidual cells are sensors for embryo health (3). In humans, endometrial stromal cells undergo decidualization during the secretory phase of the menstrual cycle, while in mice, embryo implantation is necessary for this process to occur (1). The appropriate decidualization of endometrial stromal cells requires them to proliferate, migrate, and differentiate from a fibroblast to an epithelioid-like phenotype (2). This process is under control of the ovarian steroid receptors, the estrogen receptor (ESR1) and the progesterone receptor (PGR). One of the potential coregulators of steroid receptor signaling is cyclin A2 (CCNA2).

In this issue of the *JCI*, Aljubran et al. (4) demonstrate that CCNA2 expression correlated with successful pregnancy in humans. They also showed that CCNA2 was required to support the developing embryo, using conditional ablation in mice and knockdown in a human endometrial stromal cell line.

## CCNA2 and pregnancy

Cyclins and their cyclin-dependent kinases are critical regulators of cell-cycle progression. Previous work has demonstrated that CCNA2 is a regulator of steroid hormonal signaling (5). CCNA2 has been shown to regulate the transcriptional activity of ESR1 and act as a coregulator of PGR (6–8). Aljubran et al. (4) analyzed the expression of CCNA2 in endometrial biopsies from patients undergoing in vitro fertilization prior to the procedure, during the proliferative and secretory phases of the menstrual cycle. Patients that achieved a successful pregnancy had higher levels of CCNA2 in endometrial stromal cells. Further, endometrial stromal cells from patients with higher CCNA2 showed higher levels of ESR1 and PGR expression and increased cell proliferation. These results indicate a relationship between CCNA2 levels and the ability of endometrial stromal cells to proliferate and prepare the uterus for pregnancy in response to steroid hormone stimulation. However, the relationship among ESR1, PGR, and CCNA2 was correlative and needed further experimentation to decipher the mechanism between CCNA2 and nuclear receptor signaling in the regulation of endometrial stromal cell proliferation and the ability of the uterus to support pregnancy.

**Figure 1:**
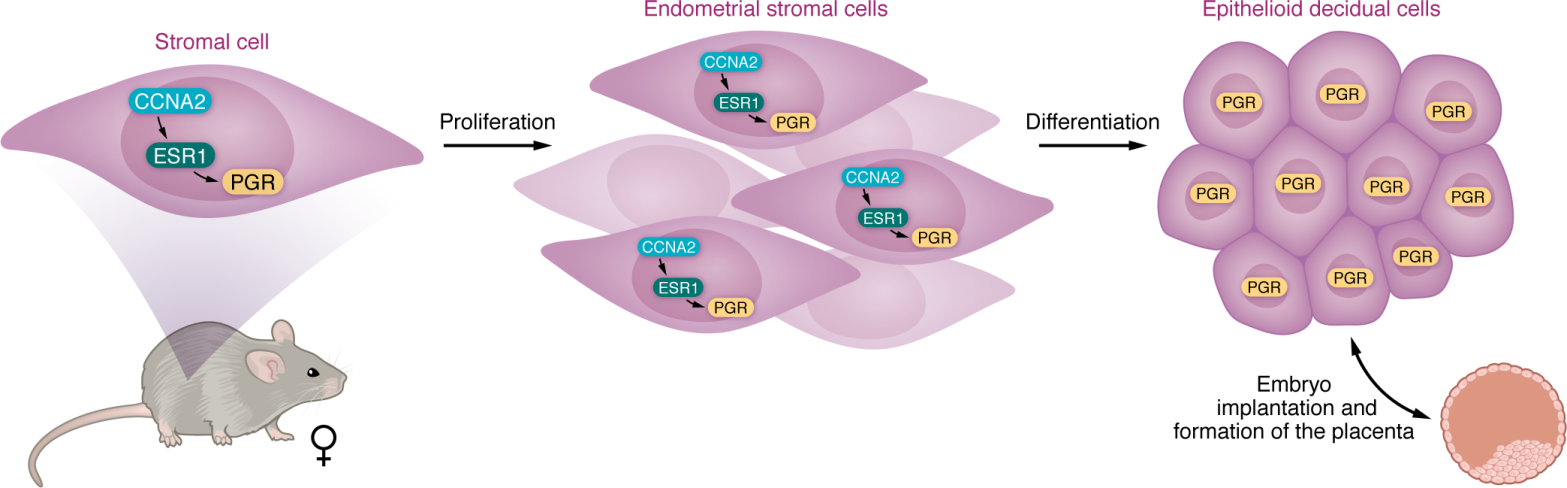
CCNA2 supports embryo implantation and embryo development. In stromal cells, CCNA2 regulates ESR1 activity, which controls PGR expression. The action of CCNA2, ESR1, and PGR results in stromal cell proliferation and their differentiation into epithelioid decidual cells. Subsequent PGR expression in decidual cells allows appropriate implantation and placentation of the embryo.

The authors examined the effects of CCNA2 deficiency to further determine the role of CCNA2 in pregnancy. They evaluated the ability of the uterus to support pregnancy and examined steroid regulation in uterine function. In a PGR^Cre^ mouse model, uterine-specific ablation of *Ccna2* resulted in infertility. The mice were able to ovulate, produce pregnancy hormones, and support attachment and implantation of embryos. However, the postimplantation survival of the embryos was impaired, resulting in embryo resorption due to the embryo’s inability to develop a functional placenta. Analysis of this phenotype demonstrated that ablation of *Ccna2* caused a hypoplastic uterus with an impaired response of the uterus to estrogen stimulation. The inability of the endometrial stromal cells to respond to estrogen resulted in the selective impairment of ESR1-regulated genes. The major ESR1 target was PGR, which showed decreased expression that resulted in an altered decidual response. The authors also used an siRNA approach to knock down CCNA2 in human endometrial stromal cells. There was a similar dependence on ESR1 regulation of PGR in the transformed cells. CCNA2 expression in human endometrial stromal cells decreased with decidualization, and CCNA2 knockdown in these cells decreased PGR expression and the ability of the cells to undergo hormonal-induced decidualization. These in vivo and in vitro results demonstrate that CCNA2 is critical for priming the endometrial stromal cells for the hormonal induction of decidualization (Figure 1).

## Summary and looking forward

CCNA2 was previously demonstrated to regulate ESR1 and PGR activity in cells in culture (5). The work by Aljubran et al. (4) demonstrates that CCNA2 regulation of steroid hormone signaling is physiologically relevant in preparing the uterus for embryo development. While most research focusing on the regulation of implantation and development of the embryo concentrates on the secretory phase of the menstrual cycle, the work by Aljubran et al. (4) encompasses the window of receptivity and demonstrates CCNA2 as an important factor during the proliferative phase of the cycle. CCNA2 during this phase is critical for priming endometrial stromal cells for hormonal induction of decidualization and giving the endometrium critical mass to support postimplantation development. Interestingly, Aljubran et al. (4) demonstrate that altered decidualization impaired the ability of the embryo to undergo placentation. While most studies focus on signaling from the trophoblasts in the placentation process, this work demonstrates that the decidua plays more than a passive role in embryo development. The in vivo model from Aljubran et al. (4) will allow further important inquiries into questions such as how the decidua regulates placentation and embryo development.

## Version 1 11/15/2024

Electronic publication
